# Health literacy and control of inflammatory activity in patients with recent-onset rheumatoid arthritis

**DOI:** 10.1093/rap/rkag049

**Published:** 2026-04-17

**Authors:** Javier Bachiller-Corral, María Auxiliadora Martin-Martínez, Diana Botello-Corzo, José L Morell-Hita, Mónica Vázquez-Díaz

**Affiliations:** Medicine, Universidad de Alcalá, Madrid, Spain; Rheumatology Department, Hospital Universitario Ramón y Cajal, Madrid, Spain; Rheumatology Department, Hospital Universitario Ramón y Cajal, Madrid, Spain; INSYCA, Madrid, Spain; Rheumatology Department, Complejo Hospitalario Universitario Insular, Las Palmas de Gran Canaria, Spain; Rheumatology Department, Hospital Universitario Ramón y Cajal, Madrid, Spain; Rheumatology Department, Hospital Universitario Ramón y Cajal, Madrid, Spain

**Keywords:** recent-onset rheumatoid arthritis, health literacy, DAS28, disease activity, health inequalities

## Abstract

**Objective:**

To determine the association between health literacy (HL) and disease activity in patients with recent-onset rheumatoid arthritis.

**Methods:**

Cross-sectional study of patients with rheumatoid arthritis (RA) of ≤ 5 years′ duration. Demographic, clinical and laboratory data were collected during routine follow-up visits. HL was assessed using the SAHLSA questionnaire, a validated measure of functional health literacy. A multivariate model was developed to evaluate the association between HL level and disease activity, measured by DAS28.

**Results:**

A total of 210 patients (81% women) were included with a mean age of 53.9 (14.1) years and a median disease duration of 1.6 [0.4–4.28] years. Low health literacy scores (SAHLSA ≤37) were identified in 10.9% (*n* = 23) of patients. These individuals showed significantly higher disease activity compared with those with higher SAHLSA scores: DAS28-ESR 3.9 (1.6) vs 2.8 (1.4) (*P* = 0.006), and DAS28-CRP 3.7 (1.4) vs 2.7 (1.2) (*P* < 0.000). Other measures—physician global assessment (VAS), tender and swollen joint counts, CRP, SDAI, CDAI, and SF-36—were also significantly worse in patients with low HL. In the multivariate analysis, compared with those with higher SAHLSA scores (OR 0.030; 95% CI 0.002–0.396; *P* = 0.008).

**Conclusion:**

Low SAHLSA health literacy scores are independently associated with poorer control of inflammatory activity in patients with RA of ≤5 years’ duration. Adapting healthcare communication and support strategies to patients’ health literacy needs early in the disease course may contribute to improved RA management and outcomes

Key messages

- 10% of the patients had inadequate health literacy, and all patients with low SAHLSA scores had moderate or high disease activity.
- Patients with lower health literacy scores showed a markedly lower probability of achieving disease control during the early years of rheumatoid arthritis.
- Direct assessment of health literacy provides more actionable information than sociodemographic indicators alone and may help tailor communication and care in early rheumatoid arthritis.

## Introduction

Health literacy (HL) is defined as the cognitive and social skills that determine the motivation and ability of individuals to access, understand, and use information to promote and maintain good health [1]. It includes key components such as reading comprehension, numeracy, and technological and social skills, and is closely related to—but distinct from—formal education.

A large body of scientific literature has demonstrated the impact of limited or inadequate health literacy on health outcomes and mortality of the general population [2]. However, despite its importance, health literacy research is often characterized by methodological limitations, which have hindered consistency, comparability and applicability of findings in clinical practice [2]. HL is considered fundamental in the management of all health conditions, but plays a particularly significant role in chronic diseases. In Spain, 54% of the population has at least one chronic disease, and this percentage is higher in people over 65 years of age [3]. For this reason, the improvement of HL is one of the objectives of the strategy for addressing chronicity in the Spanish Health System [4]. As a consequence of an inadequate HL in chronic diseases, there is an excess use of health services, which results in an inefficient system and an increased risk for the patient. In diseases such as diabetes, asthma, multiple sclerosis and cardiovascular conditions, adequate HL is associated with better disease control, reduced use of health services and lower mortality rates [5–8].

Rheumatoid arthritis (RA) is one of the most prevalent chronic inflammatory diseases in the world. RA primarily affects middle-aged women and requires continuous care in rheumatology clinics. Appropriate follow-up and adherence to medical treatment in RA are associated with reduced joint damage and better quality of life indices [9, 10].

Limited health literacy in patients with RA may contribute to delayed diagnosis and affect treatment adherence and long-term follow-up [11]. In addition, patients with inadequate HL may have difficulties filling out tools such as the visual analogue scale, potentially affecting the disease activity assessments [12]. Differences in drug prescription patterns according to health literacy level have also been observed in RA patients, and higher disease activity has been reported in patients with health literacy limitations, although not specifically in early-onset RA populations [13]. To the best of our knowledge, no studies have addressed HL impact in patients with RA during the initial years of the disease course, a period in which improving knowledge and disease management skills may be more efficient and safer. The present study aims to evaluate the association between HL and inflammatory disease control in patients with recent-onset RA (≤5 years’ duration).

## Methods

### Study design

Cross-sectional study of patients diagnosed with RA with short disease duration and treated at a hospital rheumatology department.

### Patients

We included patients aged between 18 and 80 years with a diagnosis of RA of less than five years` duration (recent-onset RA) who were regularly attending the hospital for follow-up. All patients were evaluated in a monographic arthritis clinic by four rheumatologists. The diagnosis of RA was established according to the 2010 ACR/EULAR classification criteria [14]. Patients who could not read or write, those with insufficient Spanish proficiency to complete the study assessments, and patients with severe psychiatric disorders were excluded. Over a 6-month period, all patients who met the inclusion criteria and none of the exclusion criteria were consecutively enrolled.

The study was approved by the local Ethics Committee for Clinical Research (Comité de Ética de la Investigación con medicamentos del Hospital Universitario Ramón y Cajal, Protocol No. 191-2016), and all patients received and signed a written informed consent before inclusion.

### Variables and operative definitions

The primary endpoint of the study was the proportion of patients with good control of inflammatory activity, defined as a DAS28-ESR ≤ 3.2 (rheumatoid arthritis in remission or with low activity) [15]. The main explanatory variable was the level of HL. Health literacy was assessed using the SAHLSA (Short Assessment of Health Literacy for Spanish Adults) questionnaire, a validated tool for Spanish-speaking adults [16]. This test consists of 50 common health-related terms that the patient must interpret, yielding a score from 0 to 50. One point is awarded for each term that is correctly pronounced and appropriately associated with its meaning, making the SAHLSA a performance-based measure of functional health literacy. Patients with a score of 37 or below were classified as having limited health literacy, according to the established operational definition of the SAHLSA instrument. The SAHLSA questionnaire was adapted from the REALM test [17] and was validated in a sample of 201 Spanish-speaking patients by comparing it with other health literacy measures. The SAHLSA test has good reliability and validity, can be used in clinical practice, and takes 3–6 min to complete [16]. Patients completed the test under supervision, following the tool’s recommended procedures.

Demographic, clinical and laboratory data were recorded for all patients, along with additional RA activity indices: the Simplified Disease Activity Index (SDAI) [18] and the Clinical Disease Activity Index (CDAI) [19]. In addition, patients completed the following questionnaires: (1) Health Assessment Questionnaire (HAQ) [20], (2) Short Form 36 Health Survey (SF-36) [21], (3) Functional Assessment of Chronic Illness Therapy-Fatigue (FACIT-Fatigue) [22] and (4) Simplified Medication Adherence Questionnaire (SMAQ) [23]. All these data were collected during a scheduled follow-up visit.

### Statistical analysis

Continuous variables with normal distribution were expressed as the mean and standard deviations (SD), while those with non-normal distributions were expressed as medians and interquartile ranges (IQR p25–p75). Relative frequencies and percentages were calculated for categorical variables. Missing data were handled using complete-case analysis for each variable, and denominators are specified in the corresponding tables when they differ from the total sample size.

Group comparisons were performed using parametric and non-parametric tests according to the distribution of each variable. Continuous variables were compared using the Student *t*-test or the Mann–Whitney *U* test. Qualitative variables were assessed by the Chi-squared test (with or without Yates correction), or Fisher exact test in 2 × 2 tables. A bivariate analysis was first performed to explore the association between health literacy and disease activity, as well as potential demographic and clinical covariates. A multivariable logistic regression model was then constructed to evaluate the independent association between health literacy level and inflammatory disease control, defined as DAS28-ESR ≤ 3.2. For the logistic regression, the independent variables were adjusted for potential confounders: age, sex, disease duration, autoantibody status and health-related quality of life. A forward stepwise approach was used to explore potential confounders, and variables with *P* values close to 0.20 in the bivariate analysis were included in the final multivariable model if they resulted in a change of 10% or more in the odds ratio for health literacy. Multicollinearity among candidate variables was assessed using correlation coefficients, and variables showing high collinearity were not entered simultaneously in the model. Results are presented as adjusted odds ratios (ORs) with 95% confidence intervals (CIs). All assessed potential confounders were retained in the final fully adjusted multivariable model. All analyses were performed using SPSS 26.0 (IBM Corp., 2020). IBM SPSS Statistics for Windows (Version 26.0) [Computer software].

The required sample size to study the association of inflammatory activity and HL in patients with recent-onset RA has not been clearly defined. Therefore, we calculated the statistical power for a sample of 210 subjects. Using the Granmo calculator, with an alpha risk of 0.05 in a bilateral contrast with 23 patients in the low HL group and 187 in the adequate HL group, the statistical power of the hypothesis contrast was 75% to detect a significant difference in the Disease Activity Score in 28 joints using erythrocyte sedimentation rate (DAS28-ESR) [15] of both groups. To achieve a stable multivariate model with 10 variables, at least 100 patients (10 per variable) were required, which was well below the number of patients recruited, ensuring adequate power to meet the study’s primary objective.

## Results

A total of 210 patients with recent-onset RA were included. The mean age was 53.9 (SD 14.1) years, the majority were women (80.5%), and the median disease duration was 1.6 years [IQR: 0.4–4.3]. Thirty-eight percent of the patients had been diagnosed with RA within the last 12 months, and 17% (35) were included on the day of the RA diagnosis. Seventy-seven percent had a positive rheumatoid factor, and 85% had a positive anti-citrullinated protein antibody result. At the time of inclusion, patients presented with moderate RA inflammatory activity with a mean DAS28-ESR of 2.9 (1.4) and a mean HAQ score of 0.37. Out of the total patients, 23 (10.9%) scored ≤37 on the SAHLSA test and were classified as having limited health literacy according to the SAHLSA criteria. Patients classified as having limited health literacy were predominantly foreign-born, non-Caucasians, and had a lower level of education (Table 1). Additionally, these patients showed significantly higher disease activity (mean DAS28-ESR 3.9 vs 2.8; *P* = 0.006; Table 1), including a greater number of tender and swollen joints, elevated ESR and CRP, and higher RA activity indices compared with patients with adequate HL. Health-related quality of life measured by SF-36 was also significantly lower in the low HL group. These differences were statistically significant, as shown in Table 1.

**Table 1:** Sociodemographic, clinical and laboratory characteristics of RA patients according to health literacy.

Variables	Total	Low literacy	Adequate literacy	*P*-value
	*n* = 210	*n* = 23	*n* = 187	
SAHLSA test, mean (SD)	43.5 (4.7)	33.5 (3.96)	44.8 (3.14)	0.001
Age at baseline (years), mean (SD)	53.9 (14.1)	52.3 (15.17)	54.1 (13.99)	0.55
Sex, female (%)	80.5%	82.6%	80.2%	0.78
Body mass index (kg/m^2^), mean (SD)	26.3 (4.9)	25.3 (4.15)	26.4 (5.03)	0.32
Country of Birth (Spain), (%)	69.4%	26.1%	74.7%	0.001
Race, (%)				0.001
Caucasian	81.2%	41.2%	85.5%	
Others^a^	17.7%	58.8%	14.5%	
Education level, (%)				0.000
Primary education	25.1%	82.6%	17.9%	
Secondary education	46.4%	13.0%	50.5%	
Higher education (university)	28.5%	4.3%	31.5%	
Time since diagnosis (years), median [p25–p75]	1.6 [0.4–4.3]	1.29 [0.1–2.2]	1.7 [0.4–4.4]	0.19
RF positive (%)	76.9%	100%	74.1%	0.005
ACPA positive (%)	85.4%	95.7%	84.2%	0.14
ANA positive (%)	34.6%	38.8%	34.1%	0.68
Patients VAS, median [p25–p75]	30 [10–60]	40 [20–70]	30 [10–60]	0.295
Physician VAS, median [p25–p75]	20 [10–40]	40 [20–60]	20 [10–40]	0.004
TJC, median [p25–p75]	0 [0–2]	3 [1–6]	1 [0–3]	0.004
SJC, median [p25–p75]	1 [0–3]	2 [0–4]	0 [0–2]	0.001
ESR, median [p25–p75]	9 [6–18]	15.5 [5.2–32.7]	9 [6–18]	0.139
CRP (mg/l), median [p25–p75]	3.6 [1.9–7.7]	7.4 [3.4–17.3]	3.5 [1.9–7.7]	0.039
DAS28-ESR, mean (SD)	2.9 (1.4)	3.9 (1.6)	2.8 (1.4)	0.006
DAS28-ESR ≤3.2 (%)	63.0%	37.5%	65.6%	0.027
DAS28-CRP, mean (SD)	2.9 (1.2)	3.7 (1.4)	2.7 (1.2)	0.000
SDAI, median [p25–p75]	6.4 [3.2–14.8]	16.5 [7.1–26.2]	6.4 [3–13.2]	0.038
CDAI, median [p25–p75]	6 [3–13.2]	16 [7–23]	6 [3–13.2]	0.009
HAQ, median [p25–p75]	0.37 [0.1–0.8]	0.6 [0.1–1.5]	0.37 [0.1–0.8]	0.300
FACIT-*F* scale, mean (SD)	31.4 (15.6)	28.7 (17.4)	31.7 (15.4)	0.38
SMAQ adherence questionnaire, (%)				0.18
Adherent to treatment	82.9%	95.7%	81.3%	
Non-adherent to treatment	7.6%	4.3%	8.0%	
SF-36, mean (SD)	60.1 (20.1)	56.5 (25.4)	66.4 (20.5)	0.03

ACPA: anti-citrullinated peptide antibodies; ANA: antinuclear antibodies; BMI: body mass index (kg/m^2^); CDAI: clinical Disease Activity Index; CRP: C-reactive protein; DAS28: disease activity score (28 joints); ESR: erythrocyte sedimentation rate (mm/h); FACIT-F: Functional Assessment of Chronic Illness Therapy—Fatigue; HAQ: Health Assessment Questionnaire; RF: rheumatoid factor; SAHLSA: Short Assessment of Health Literacy for Spanish-speaking Adults; SD: standard deviation; SDAI: simplified disease activity index; SF-36: Short Form 36 Health Survey Questionnaire; SJC: swollen joint count; SMAQ: Simplified Medication Adherence Questionnaire; TJC: tender joint count; VAS: global health visual analogue scale.

a The ‘Other’ category includes participants of ‘Latin American, North African, Asian, and mixed ethnic backgrounds’. Percentages were calculated using complete-case analysis for each variable. The number of observations for each variable may differ from the total sample (*n* = 210) due to missing data. Minor discrepancies are due to rounding.

A bivariate analysis was performed to determine differences according to sociodemographic variables (age, sex, education level, and country of birth), clinical and quality-of-life outcomes among patients with rheumatoid arthritis. The stratified analysis by disease activity level showed patients with poor inflammatory control had significantly lower SAHLSA scores and worse clinical, laboratory, functional and quality of life outcomes. Disease duration was shorter in patients with poor disease control (median 0.8 vs 2.2 years; *P* = 0.031; Table 2). Further variables analyzed are presented in Table 2. In the multivariate logistic regression analysis evaluating the association between HL and inflammatory activity, low HL was significantly associated with poor RA control (OR: 0.03; 95% CI: 0.002–0.396; *P* = 0.008), even after adjustment for potential confounders: age, sex, disease duration, autoantibody status and health-related quality of life (Table 3). Additionally, the presence of positive ACPA (OR: 0.260; 95% CI: 0.071–0.955; *P* = 0.042) and higher SF-36 scores (OR: 0.918; 95% CI: 0.886–0.952; *P* = 0.001) were also independently associated with worse control of inflammatory activity. Although disease duration was associated with inflammatory activity in the bivariate analysis, this association did not remain statistically significant in the fully adjusted model. Country of birth was excluded from the final multivariable model due to multicollinearity with other sociodemographic variables.

**Table 2:** Sociodemographic, clinical and laboratory characteristics of RA patients according to DAS28-ESR.

Variables	DAS28-ESR ≤ 3.2	DAS28-ESR > 3.2	*P*-value
	*n* = 109	*n* = 64	
SAHLSA test, mean (SD)	44.35 (3.79)	42.69 (5.43)	0.019
Age at baseline (years), mean (SD)	53.9 (12.9)	52.21 (16.5)	0.453
Sex, female (%)	77.1 %	89.1%	0.05
Body mass index (kg/m^2^), mean (SD)	25.9 (4.7)	26.2 (4.9)	0.744
Country of birth (Spain) (%)	73.4%	64.1%	0.196
Race, (%)			0.048
Caucasian	86.7%	74.1%	
Other	13.3%	25.9%	
Education level, (%)			0.106
Primary education	18.9%	32.8%	
Secondary education	50.9%	39.1%	
Higher education (university)	30.2%	28.1%	
Time since diagnosis (years), median [p25–p75]	2.2 [0.8–4.57]	0.8 [0.11–3.9]	0.031
RF positive (%)	80%	77.4%	0.606
ACPA positive (%)	89.8%	82.5%	0.170
ANA positive (%)	35%	37.5%	0.755
ESR (mm/h), median [p25–p75]	7 [4.5–12]	25 [14–40]	0.001
CRP (mg/l), median [p25–p75]	2.8 [1.5–5.2]	6.8 [3.5–14.6]	0.001
DAS28-CRP, mean (SD)	2.7 (1.2)	3.7 (1.4)	0.000
SDAI, median [p25–p75]	4.09 [2.3–7.08]	19.5 [14.24–24.62]	0.001
CDAI, median [p25–p75]	3.4 [2–6]	18 [13–24]	0.001
HAQ, median [p25–p75]	0.25 [0.25–0.62]	0.93 [0.28–1.37]	0.001
FACIT-F scale, mean (SD)	32.4 (17.8)	29.3 (13.2)	0.248
SMAQ adherence questionnaire, (%)			0.652
Adherent to treatment	88.4%	81.3%	
Non-adherent to treatment	7.3%	6.3%	
SF-36, mean (SD)	69.07 (14.24)	47.6 (19.73)	0.001

ACPA: anticitrullinated peptide antibodies; ANA: antinuclear antibodies; CDAI: Clinical Disease Activity Index; CRP: C-reactive protein; DAS28: disease activity score (28 joints); ESR: erythrocyte sedimentation rate (mm/h); FACIT-F: Functional Assessment of Chronic Illness Therapy—Fatigue; HAQ: Health Assessment Questionnaire; RF: rheumatoid factor; SAHLSA: Short Assessment of Health Literacy for Spanish-speaking Adults; SD: standard deviation; SDAI: Simplified Disease Activity Index; SF-36: Short Form 36 Health Survey Questionnaire; SMAQ: Simplified Medication Adherence Questionnaire.

**Table 3:** Multivariable logistic regression analysis of factors associated with controlled inflammatory activity (DAS28-ESR ≤ 3.2).

Variables	OR_ajusted_	95% CI	*P*-value
SAHLSA (ref. low literacy)	0.030	0.002–0.396	0.008
Age, years	1.004	0.966–1.043	0.848
Sex (ref. female)	1.018	0.246–4.211	0.980
ACPA (ref. negative)	0.260	0.071–0.955	0.042
Time since diagnosis, years	0.815	0.623–1.066	0.135
SF-36	0.919	0.886–0.952	0.001

ACPA: anticitrullinated peptide antibodies; DAS28: disease activity score (28 joints); ESR: erythrocyte sedimentation rate (mm/h); HL: health literacy; RA: rheumatoid arthritis; SAHLSA: Short Assessment of Health Literacy for Spanish-speaking Adults; SF-36: Short Form 36 Health Survey Questionnaire. Low health literacy, defined as SAHLSA ≤37 was used as the reference category.

## Discussion

The main objective of this study was to determine the association between HL and the control of inflammatory activity in recent-onset RA. Our findings show a strong association between limited health literacy, as operationalized by the SAHLSA instrument, and elevated RA inflammatory activity, even in early RA stages. The multivariate model indicated that patients with limited health literacy had a markedly lower probability of achieving disease control (OR 0.03), compared with those with adequate HL, representing a 97% reduction in the probability of disease control in the low HL group. The median disease duration in our sample was 1.6 years and therefore includes patients with a short course of the disease. All disease activity parameters were significantly higher in patients with low health literacy, suggesting that health literacy may influence disease management during diagnosis and treatment initiation.

The SAHLSA test is a useful performance-based tool to identify functional health literacy limitations in rheumatology consultations. In our study, 11% of patients were classified as having inadequate HL—lower than the rates reported in other studies. Gordon *et al.* [17] found a prevalence of 15%, while a more recent study by Gorter *et al.* [13]. reported that 19.4% of RA patients were classified in the lowest health literacy category, while a larger proportion showed some degree of health literacy limitation. However, these comparisons should be interpreted with caution due to differences in the instruments used to measure HL.

Importantly, health literacy should not be understood exclusively as an individual attribute or deficit. In this study, health literacy was assessed using the SAHLSA, a tool that measures functional health literacy related to the comprehension of general health-related terms, rather than disease-specific knowledge or patients’ understanding of rheumatoid arthritis itself.

From a measurement perspective, it is important to acknowledge that different health literacy instruments capture distinct dimensions of this construct. According to Nutbeam’s framework [24], the SAHLSA assesses functional health literacy, but does not capture interactive or critical dimensions related to patient engagement or critical appraisal of health information.

Therefore, our findings should not be interpreted as reflecting patients’ knowledge of RA, but rather their ability to process and use health information within healthcare settings.

From this perspective, inadequate health literacy may act as a barrier to effective communication, shared decision-making, and engagement with healthcare services, particularly in the early stages of the disease. Addressing health literacy needs should thus involve not only efforts aimed at supporting individual skills, but also adaptations at the healthcare system level, including clearer communication, simplified information materials, and health literacy–responsive care environments.

In our cohort, patients with limited health literacy had significantly lower educational attainment and differed racially, with a lower proportion of Caucasians and a higher representation of participants from other ethnic backgrounds. While both low education level and inadequate HL have been associated with poor prognosis in RA, they are not synonymous. Buchbinder *et al.* [25] demonstrated a strong correlation between educational level and HL, but HL-specific tests allow patients to be classified more accurately. In addition, Caplan *et al.* [26] found that HL was more strongly associated with long-term functional impairment (measured by the HAQ) than education.

There is strong scientific evidence supporting the development of disease-specific health literacy assessment tools, and health literacy instruments have already been adapted for diseases such as diabetes [27] and multiple sclerosis [28]. No specific tool has currently been developed or validated for RA to date, and future studies in this field are necessary.

Interestingly, in our study, patients with inadequate health literacy did not demonstrate worse pharmacological adherence compared with those with adequate literacy. Similar findings have been reported in other studies [29], though contrasting results exist showing reduced pharmacological adherence in patients with low HL [11]. The discrepancies between studies may be attributable to the use of different adherence measurement tools and the potential influence of hospital staff assisting patients during questionnaire completion.

Patients with inadequate HL also showed poorer health-related quality of life parameters (as measured by SF-36) compared with those with adequate HL. In contrast, no significant differences were observed in indicators of chronic damage, such as functional impairment measured by the HAQ. This finding differs from previous studies [26, 30], which reported evident declines in functional capacity among patients with inadequate HL. This discrepancy is likely due to the short RA duration in our cohort (median of 1.6 years), where significant functional or chronic damage had not yet developed, whereas in the study by Caplan *et al.* [26], the mean course of the disease was greater than 17 years.

Limitations of the study include the relatively small sample of patients with inadequate HL. Even though the statistical power achieved with 210 subjects was 75%, the results were robust, the multivariate model included a limited number of independent variables and accuracy could be ensured. Given the cross-sectional design, we could identify associations but not establish causality. Longitudinal studies are necessary to explore causal relationships, followed by interventional studies to assess the impact of HL improvement on the control of disease activity. The representativeness of the study sample should be considered when interpreting these findings. Although patients were recruited consecutively from a routine early rheumatoid arthritis clinic and informed consent was obtained through face-to-face explanations, it is possible that individuals with more pronounced health literacy challenges were less likely to participate. No adapted information materials or patient research partner involvement were incorporated into the recruitment strategy, which may have limited inclusiveness. Future studies should consider implementing health literacy–responsive recruitment approaches.

Moreover, the use of a performance-based instrument such as the SAHLSA may have influenced participation, as such assessments can evoke a sense of being ‘tested’, potentially discouraging engagement among patients with lower literacy skills. Together, these factors may have contributed to selection bias and to an underestimation of the prevalence of limited health literacy in this cohort. Consequently, the observed associations should be interpreted with caution, particularly with regard to their generalizability to the broader population of patients with early rheumatoid arthritis.

Additionally, we were unable to evaluate the impact of inadequate HL on healthcare resources utilization, such as increased consultations or diagnostic tests and medication use. This is a key area to maintain the sustainability and efficiency of the health system since longitudinal studies [13] have shown that improved HL is associated with reduced healthcare utilization, enhanced patient self-management of the disease, reduced risks for the patients and, ultimately, an improvement in the efficiency of the system. Health literacy should be understood as a multidimensional and context-dependent construct, and the classification used in this study reflects an operational definition for analytical purposes rather than a fixed or intrinsic characteristic of individuals [31].

In conclusion, more than 10% of patients with recent-onset rheumatoid arthritis in our sample were classified as having limited health literacy according to a validated assessment tool. Limited health literacy was independently associated with poorer disease activity control. Adapting healthcare communication and support strategies to patients’ health literacy needs is particularly important in the early years following RA onset. Further research is needed to develop disease-specific tools to measure health literacy in rheumatoid arthritis and to assess the causal effects of limited health literacy on disease control and healthcare system sustainability. Moreover, interventional studies should evaluate strategies aimed at improving health literacy and patient education in rheumatoid arthritis. These findings highlight the importance of recognizing health literacy needs early in the disease course.

## Data Availability

The data underlying this article will be shared on reasonable request to the corresponding author.
